# Editorial: Advanced therapies for cardiac regeneration, volume II

**DOI:** 10.3389/fbioe.2026.1861887

**Published:** 2026-05-28

**Authors:** Monica Boffito, Susanna Sartori, Valeria Chiono, Irene Carmagnola

**Affiliations:** Department of Mechanical and Aerospace Engineering, Politecnico di Torino, Turin, Italy

**Keywords:** cardiac tissue engineering/regenerative medicine, hydrogel, imaging, nanomedicine, reprogramming, scaffold, stem cells, tissue model

Cardiac regeneration remains a major clinical challenge with significant social and economic impact. Multidisciplinary approaches are increasingly used to better understand the causes and progression of cardiac diseases, improving their diagnosis and treatment. In recent years, cardiac tissue engineering has made notable progress, driven by the integration of bioengineering, advanced imaging, and regenerative medicine.

This Research Topic gathers original articles, presenting next-generation tools—ranging from advanced imaging systems and bioreactors to myocardial slices and bioengineered scaffolds—enabling precise, physiologically relevant, and scalable approaches to cardiac repair. These editorial summarizes these contributions and highlights their key findings.

Two research articles report on the great potential of real-time and high-resolution imaging for the functional evaluation of cardiac tissues models. These two imaging studies—one *ex vivo* and one *in vivo*—illustrate the transformative role of advanced optical technologies. From the engineered monolayer to the living myocardium, high-resolution imaging is redefining how cardiac physiology and pathology can be quantified and optimized.

In detail, the work by Belzil et al. introduces a lensless, high-speed imaging platform integrated with electrical stimulation for the evaluation and monitoring of cardiac engineered tissues (Belzil et al.). The system enables label-free, quantitative assessment of contractile behavior and cellular organization in stem cell-derived cardiac monolayers. Its ability to capture structural and functional parameters makes it a valuable tool for standardizing differentiation protocols and optimizing culture conditions. Validation with human induced pluripotent stem cell (iPSC)-derived and neonatal rat cardiomyocytes confirms its robustness. This imaging approach overcomes limitations of conventional microscopy, offers high temporal resolution, and links functional monitoring with improved cardiac tissue maturation.

The second original article focused on imaging techniques pioneers *in vivo* subcellular imaging of the beating heart (Kuo et al.). By synchronizing two-photon microscopy with electrocardiographic signals, the authors enabled long-term, high-precision imaging of the contracting myocardium. They identified sarcomeric changes linked to functional impairment, providing insight into post-infarction remodeling. This platform allows real-time evaluation of nanoscale cardiac dynamics and *in situ* monitoring of cytoskeletal and T-tubule remodeling, highlighting the role of structural integrity in contractile function and supporting the validation of biomimetic therapies.

Two articles propose tissue engineering approaches for the development of cardiovascular constructs. These works illustrate diverse strategies—from biologically seeded grafts with intrinsic regenerative capacity to structurally engineered scaffolds—aimed at emulating the physical and mechanical cues of native tissues, thereby enabling new tissue formation for cardiovascular repair.

For instance, the study by Iacobazzi et al. provides preclinical evidence supporting the development of living, self-remodeling vascular conduits. Using a porcine model, the authors compared small intestinal submucosa-derived grafts seeded with Wharton’s Jelly derived mesenchymal stromal cells (WJ-MSCs) *versus* acellular controls for main pulmonary artery replacement. Over 6 months, WJ-MSC-seeded grafts exhibited superior remodeling, increased elastin content, and preserved patency in absence of thrombosis or calcification. These findings suggest that WJ-MSCs can turn passive scaffolds into living tissues that grow and repair, overcoming the long-term limits of current prosthetics.

The other research article investigates biofabrication strategies for engineering architecturally complex cardiac scaffolds (Ketabat et al.). The authors developed alginate/gelatin constructs with optimized properties, identifying one formulation with the best balance of elasticity, swelling, and biocompatibility. A novel angular scaffold design improved cell viability and organization over traditional lattices, advancing replication of the myocardium’s anisotropic and hierarchical architecture.

Proper recapitulation of the native myocardial microenvironment, particularly in terms of biochemical cues, is a key factor in cardiac tissue engineering. In this Research Topic Ruocco et al. investigated how extracellular matrix (ECM) proteins modulate used four-microRNA cocktail (miRcombo) and miRcombo-mediated reprogramming of adult human cardiac fibroblasts (AHCFs). Specifically, the authors demonstrated that ECM protein composition influences miRcombo-mediated direct reprogramming of AHCFs by modulating cell behavior, cytoskeletal organization, and mechanosensitive signaling pathways. Among the tested proteins, laminin and an AHCF-produced decellularized ECM provided the most supportive microenvironment for cardiac marker expression and structural maturation, whereas fibronectin and collagen I were associated with lower reprogramming efficiency and a more proliferative phenotype.

The remaining two papers highlight the necessity of reliable *in vitro* models for advancing fundamental research and evaluating the efficacy of new cardiac therapies.

Nunez-Toldra et al. proposed the use of living myocardial slices (LMS) as *ex vivo* translational models for gene therapy research (Nunez-Toldra et al.). In their work, thin, viable slices of rat and human cardiac tissue were maintained under physiological sarcomere length and electrical stimulation, and the protocol for their efficient transduction using some of the most cardiotropic adeno-associated viral vectors was optimized. Furthermore, LMS derived from failing human hearts were employed to validate the translational potential of the platform. This work thus established living myocardial tissue as a physiologically relevant substrate for preclinical testing of gene-based interventions, facilitating the transition from rodent studies to human-relevant models.

In the other work, Takaya et al. investigated the role of eletromechanical cues in producing *in vitro* cardiac tissue models and disease simulation (Takaya et al.). The authors developed BEaTS-β, an open-source electromechanical bioreactor that integrates adjustable mechanical stretch, electrical pacing, and hypoxic control to mimic pathophysiological conditions such as myocardial infarction and arrhythmia. Human iPSC-derived cardiomyocytes, fibroblasts, and endothelial cells cultured under these conditions demonstrated decreased maturation and survival, faithfully mimicking clinical outcomes. Furthermore, the open-source nature of the system ensures reproducibility and encourages collaborative refinement, effectively mitigating the challenge of establishing standardized, physiologically relevant disease models. BEaTS-β can thus bridge the gap between static cultures and dynamic organ-level behavior, offering a controllable environment for mechanobiological and pharmacological investigations.

Altogether, BEaTS-β and LMS exemplify the emergence of dynamic, biomimetic cardiac models that faithfully recapitulate physiological and pathological cues. Their integration with imaging and biosensing systems represents a decisive step toward fully functional, humanized cardiac testbeds.

The collective contribution of all the seven studies of this Research Topic lies not merely in their individual innovations but in their synergistic integration, essential for achieving predictive and translational products. Soon, automated imaging could guide scaffold design and maturation in real time, while patient-derived iPSCs could populate 3D-printed constructs subjected to personalized mechanical and electrical conditioning—all monitored through lensless or intravital imaging systems.

